# Spatial-Temporal Relationship Between Population Mobility and COVID-19 Outbreaks in South Carolina: Time Series Forecasting Analysis

**DOI:** 10.2196/27045

**Published:** 2021-04-13

**Authors:** Chengbo Zeng, Jiajia Zhang, Zhenlong Li, Xiaowen Sun, Bankole Olatosi, Sharon Weissman, Xiaoming Li

**Affiliations:** 1 South Carolina SmartState Center for Healthcare Quality Arnold School of Public Health University of South Carolina Columbia, SC United States; 2 Department of Health Promotion, Education, and Behavior Arnold School of Public Health University of South Carolina Columbia, SC United States; 3 Big Data Health Science Center University of South Carolina Columbia, SC United States; 4 Department of Epidemiology and Biostatistics Arnold School of Public Health University of South Carolina Columbia, SC United States; 5 Geoinformation and Big Data Research Lab Department of Geography, College of Arts and Sciences University of South Carolina Columbia, SC United States; 6 Department of Health Services, Policy, and Management Arnold School of Public Health University of South Carolina Columbia, SC United States; 7 School of Medicine University of South Carolina Columbia, SC United States

**Keywords:** COVID-19, mobility, incidence, South Carolina

## Abstract

**Background:**

Population mobility is closely associated with COVID-19 transmission, and it could be used as a proximal indicator to predict future outbreaks, which could inform proactive nonpharmaceutical interventions for disease control. South Carolina is one of the US states that reopened early, following which it experienced a sharp increase in COVID-19 cases.

**Objective:**

The aims of this study are to examine the spatial-temporal relationship between population mobility and COVID-19 outbreaks and use population mobility data to predict daily new cases at both the state and county level in South Carolina.

**Methods:**

This longitudinal study used disease surveillance data and Twitter-based population mobility data from March 6 to November 11, 2020, in South Carolina and its five counties with the largest number of cumulative confirmed COVID-19 cases. Population mobility was assessed based on the number of Twitter users with a travel distance greater than 0.5 miles. A Poisson count time series model was employed for COVID-19 forecasting.

**Results:**

Population mobility was positively associated with state-level daily COVID-19 incidence as well as incidence in the top five counties (ie, Charleston, Greenville, Horry, Spartanburg, and Richland). At the state level, the final model with a time window within the last 7 days had the smallest prediction error, and the prediction accuracy was as high as 98.7%, 90.9%, and 81.6% for the next 3, 7, and 14 days, respectively. Among Charleston, Greenville, Horry, Spartanburg, and Richland counties, the best predictive models were established based on their observations in the last 9, 14, 28, 20, and 9 days, respectively. The 14-day prediction accuracy ranged from 60.3%-74.5%.

**Conclusions:**

Using Twitter-based population mobility data could provide acceptable predictions of COVID-19 daily new cases at both the state and county level in South Carolina. Population mobility measured via social media data could inform proactive measures and resource relocations to curb disease outbreaks and their negative influences.

## Introduction

Since the first confirmed case of COVID-19 in the United States on January 21, 2020, countrywide COVID-19 outbreaks have surged. As of March 5, 2021, there were 28,580,198 cumulative confirmed cases and 517,224 COVID-19–related deaths in the United States [1]. South Carolina, a state located in the southeastern United States, had its first confirmed cases on March 6, 2020. From March to May 2020, the trend of daily new cases was flat, with an average daily increase in cases of less than 500. However, the daily new cases in South Carolina have risen sharply since June 2020. On July 14, 2020, COVID-19 cases in South Carolina surpassed 60,000, with more than 2200 daily new cases, the second highest increase in one day in the United States [2]. Between August and October 2020, the transmission rate slowed down with the further implementation of nonpharmaceutical interventions (NPIs), such as dine-in service restrictions and face-covering requirements, but increased steadily after October. By March 5, 2021, there were 448,275 reported cases and 7697 deaths in South Carolina [3].

Given the rapid transmission of COVID-19 and limited options in terms of medical interventions, forecasting is of critical importance as it could predict the spread of disease, estimate the impacts of NPIs, and inform further decision making regarding public health interventions [4]. During the COVID-19 pandemic, decision makers in the United States need to balance the net losses arising from social interruptions, economic damage, and indirect effects on health caused by NPIs with the direct health benefits of disease control [5]. Accurate and reasonable forecasting of COVID-19 could minimize the disease burden in health care settings and the loss of health and life in different phases of reopening plans [5,6].

Existing literature has suggested that population mobility may reflect the influences (both positive and negative) of NPIs, reopening actions, and public holidays [7-9]. For instance, in the early stages of the COVID-19 pandemic, the governor of South Carolina issued a series of NPIs, such as shelter-in-place and the closure of schools and nonessential businesses, to reduce social interaction. These NPIs showed positive effects in suppressing the statewide spread of COVID-19. Later, in May 2020, reopening policies and public holidays diluted the implementation of NPIs, leading to increased social interactions and statewide COVID-19 spread [10,11]. At present, it may be difficult to directly measure the real-time impact of reopening policies, public holidays, and NPI implementation fidelity. Therefore, population mobility may be a proximal indicator allowing for real-time COVID-19 transmission forecasting.

Social media platforms, such as Twitter, collect geospatial information and closely monitor changes in population mobility [12,13]. Indeed, the tremendous volume of user-generated geoinformation from social media enables the real-time or near real-time surveillance of population mobility and provides timely data on how population mobility changes in response to different phases of the COVID-19 outbreak, policy reactions, and public holidays [14-16]. Several studies have leveraged mobility data from social media (eg, Google, Facebook, Twitter) to investigate the relationship between population mobility and COVID-19 transmission [9,11,17-19]. These studies identified a consistently positive relationship between population mobility and COVID-19 incidence. However, few studies used population mobility as a predictor to forecast further outbreaks and to evaluate prediction accuracy in addition to performing correlation analysis. A study by Wang and Yamamoto [19] predicted COVID-19 daily new cases in Arizona using disease surveillance data, the Google Community Mobility report, and partial differential equations. They found an acceptable prediction accuracy for the next 3 days, but the time window of prediction did not cover the duration of viral incubation (ie, 14 days). Furthermore, this study only split Arizona into three regions (ie, central, northern, and southern) rather than examining prediction accuracy at both the state and county level. In fact, there may be geospatial differences in population mobility due to the plausible differential implementation fidelity of NPIs and reactions to reopening policies by county [20,21]. Additionally, there may be geospatial differences in the estimation of population mobility on social media as the number of users and their demographic characteristics may differ by county. All these differences may result in variations in prediction accuracy at the county level, and further studies are needed in this regard.

Prior research has predicted COVID-19 incidence using disease surveillance data and several different time series methods. Most of the studies successfully incorporated the association of the current incidence with the previous incidence using time series methods such as autoregressive, moving average, autoregressive integrated moving average (ARIMA), and Holt-Winters [22]. Some studies used generalized linear regression with continuous outcomes (eg, rate and count), without including time series [23]. However, there were few studies that simultaneously considered time-varying population mobility. Recently, Liboschik and colleagues [24] suggested that count time series following generalized linear models could overcome the limitations of classic time series methods. Based on the generalized linear model methodology, a suitable distribution for count data and appropriate link function could be specified, and the effect of the time-varying covariate could be tested and integrated into forecasting. In this study, we adopted the Poisson count time series model and time-varying population mobility data extracted from Twitter, which may increase the accuracy of COVID-19 prediction.

To address these knowledge gaps, by leveraging disease surveillance data and Twitter-based population mobility, this study aimed to construct Poisson count time series models of COVID-19 daily new cases, investigate the relationship between them, and evaluate the prediction accuracy of daily new cases for the next two-week window at both the state and county level in South Carolina.

## Methods

### COVID-19 Incidence Data

Cumulative confirmed cases of COVID-19 through November 11, 2020, at both the state and county level in South Carolina were collected from The New York Times data set, which was deposited in GitHub [25]. The data set was compiled using data from state and local governments and health departments, ensuring its accuracy. Within the study period (March 6, 2020 [date of first COVID diagnosis in South Carolina] to November 11, 2020 [251st day]), daily new cases were calculated by subtracting the cumulative confirmed cases of the previous day from the total cases for the entire state and its five counties with the largest numbers of cumulative confirmed cases (ie, Charleston, Greenville, Horry, Spartanburg, and Richland). The study protocol was approved by the Institutional Review Board at the University of South Carolina.

### Population Mobility

Population mobility was determined using the number of people (Twitter users) with a moving distance greater than 0.5 miles per day in South Carolina and the selected counties. The methodology of extracting daily population movement (origin-destination flows) from geotagged tweets is discussed elsewhere [26,27]. Briefly, geotagged tweets during the study periods were collected and used for calculation. Only users who posted at least twice per day or posted tweets on at least two consecutive days were included in the calculation. Daily travel distance was calculated for each user based on the derived origin-destination flows and used to generate a variable of how many people moved each day (with a travel distance greater than 0.5 miles). This method of capturing population mobility using Twitter has been previously validated [16,26].

### Statistical Analysis

First, daily new cases of COVID-19 and population mobility at both the state and county level were described using line charts in R (version 3.6.3; R Foundation for Statistical Computing; “ggplot” package). Daily new cases and mobility were also described using five quantiles (ie, minimum, 25th percentile, 50th percentile, 75th percentile, and maximum) for each month.

Second, a Poisson count time series model was used to model the impact of population mobility on the daily new cases of COVID-19 at the state level. Time series models were built at various time windows. For the first-round selection, a total of 17 time windows (by 7-day increments) were considered, including 1-7 days, 1-14 days,…, and 1-119 days. The daily new cases from the first to the 234th day were used as the training data set, and those from the next 3 days (days 235-237) were used as a testing data set for the purpose of model evaluation. With the smallest prediction error (equation 1) and good interpretation, the predictive model with the best time window was selected. After the best time window in the first round selection was determined, second- and third-round selections were conducted to narrow down the time window and obtain the final model with the smallest prediction error. The final model was used to predict the COVID-19 daily new cases for the next 3, 7, and 14 days (days 238-251). The cumulative difference (equation 2) between observed and predicted cases and mean absolute percentage accuracy (equation 3) for each time frame were reported [19]. The equations used are as follows:


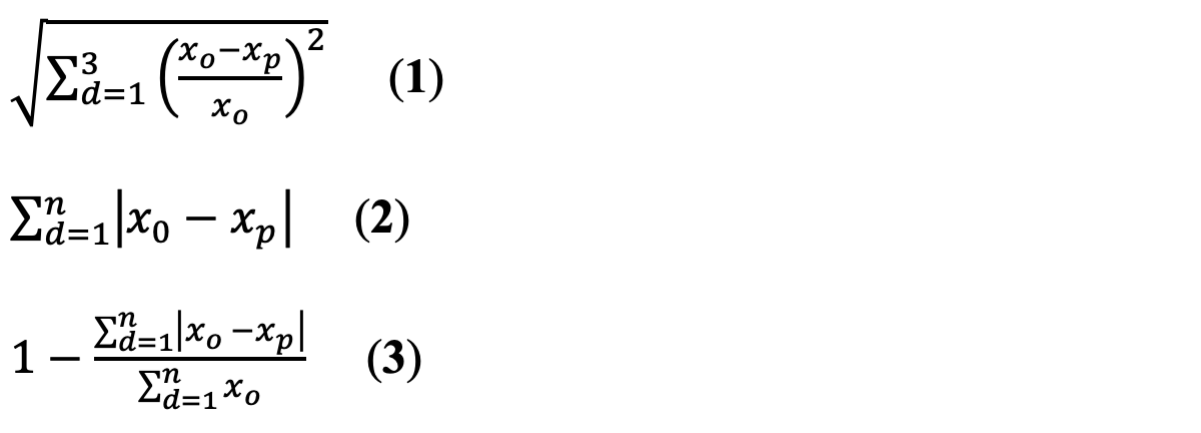


In equations 1-3, *d* represents the day; *n* is the next 3, 7, or 14 days; *o* is the observed value, *p* is the predicted value, and *x* represents the daily new cases.

Finally, a similar analytic procedure was performed to construct the final model at the county level for each of the top five counties (ie, Charleston, Greenville, Horry, Spartanburg, and Richland) in South Carolina. A Poisson count time series model was conducted using an R package (“tscount”). Table S1 in Multimedia Appendix 1 provides a detailed description of the data acquisition process, scripts for analysis and figures, and a link to data resources.

## Results

### Descriptive Statistics

**Figure 1:**
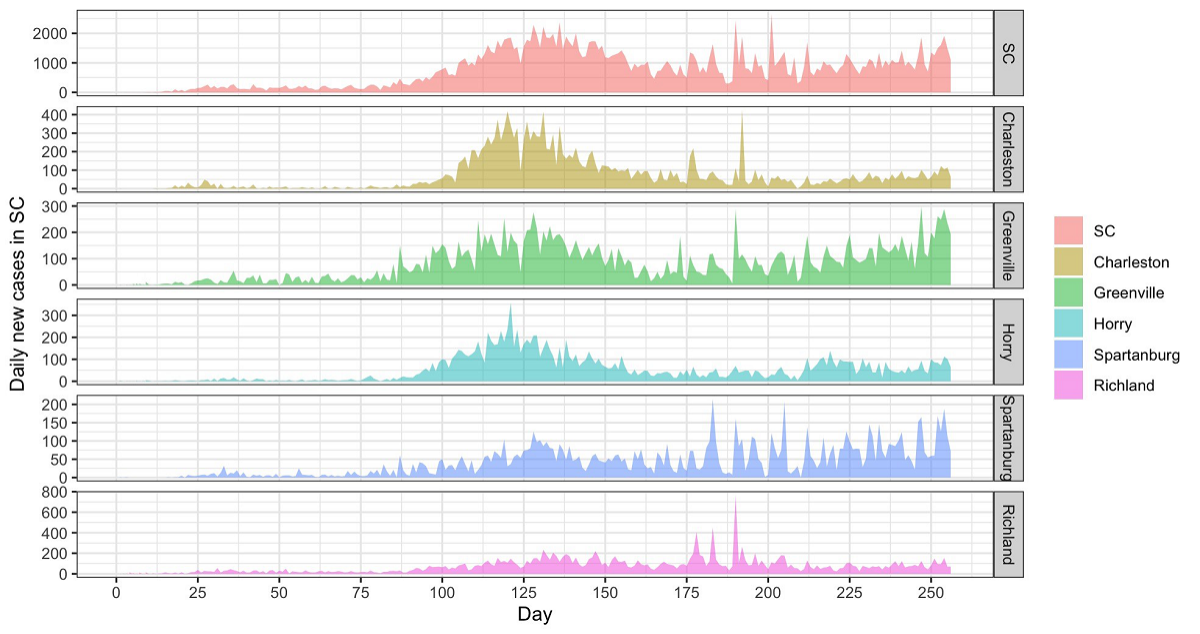
Daily COVID-19 new cases at both state and county level in South Carolina. SC: South Carolina.

Figure 1 shows the changes in COVID-19 daily new cases at both the state and county level. By October 31, 2020, there were 176,612 cumulative confirmed COVID-19 cases in South Carolina. The cumulative confirmed cases in Charleston, Greenville, Horry, Spartanburg, and Richland were 17,384, 18,021, 12,591, 9290, and 17,531, respectively. At the state level, the daily new cases from March to the end of May were less than 500. From June to the middle of July, the number of daily new cases rose, with 2217 new patients with confirmed COVID-19 on July 14. After that, the transmission rate decreased, with most daily new case counts staying under 1500. However, since October 2020, the daily new cases have steadily increased.

At the county level, the top five counties showed a similar trend of COVID-19 outbreaks and accounted for more than 40% of the total cases in South Carolina. The daily new cases increased earlier in Greenville than in the other four counties (ie, Charleston, Horry, Spartanburg, and Richland).

**Figure 2:**
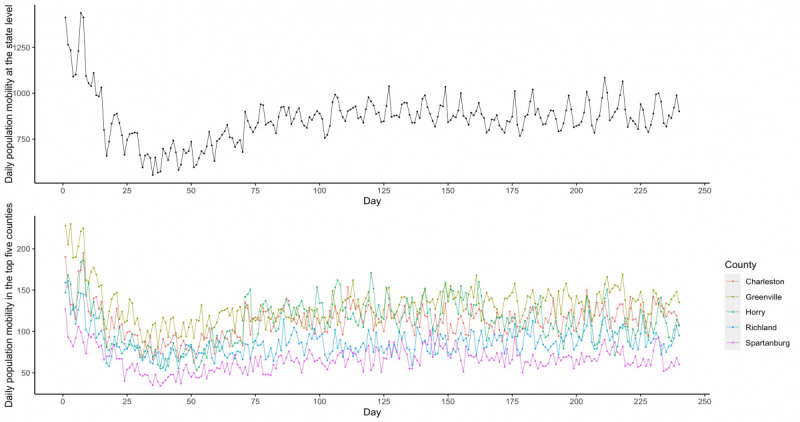
Daily population mobility at both state and county level in South Carolina.

Trends for population mobility at both the state and county level were similar. The number of people in South Carolina (Twitter users in our data) with a moving distance of more than 0.5 miles decreased from 1400 to 550 between March 6 and April 9, 2020. Although there were slight increases from the middle of April to that of June, the numbers were consistently around 1000 after this timeframe. At the county level, each of the five counties had less than 200 people with a moving distance greater than 0.5 miles after the middle of March. Figure 2 shows the changes in population mobility at both the state and county level. Table S2 in Multimedia Appendix 1 presents the descriptive statistics of population mobility and COVID-19 new cases at both the state and county level.

### Model Selection of Time Series Analyses

Following the model selection procedure, a Poisson count time series model of COVID-19 incidence at the state level was constructed using daily new cases and population mobility. Population mobility was positively associated with state-level COVID-19 daily new cases (*β*=.818, 95% CI .761-.876), and the model using the past 7 days (1-7 days) as the time window had the smallest prediction error (Table 1). The prediction error of new cases in the next 3 days (days 235-237) was 0.294.

At the county level, a similar modelling procedure was employed. Population mobility was consistently and positively associated with new cases of COVID-19 across the top five counties. The best time windows for Charleston, Greenville, Horry, Spartanburg, and Richland were 9, 14, 28, 20, and 9 days, respectively. Table 1 displays the detailed results of the final model, the correlation analysis, and the 3-day prediction error at both the state and county level.

**Table 1:** The impacts of population mobility on COVID-19 outbreaks in South Carolina.

Parameters			State level		County level								
					Charleston		Greenville		Horry		Spartanburg		Richland
**Model training**													
	Time windows (days)	1-7		1-9		1-14		1-28		1-20		1-9	
	Coefficient of population mobility (95% CI)	0.818 (0.761-0.876)		0.486 (0.338-0.634)		0.278 (0.165-0.390)		0.395 (0.275-0.515)		0.270 (0.118-0.422)		0.157 (0.067-0.246)	
	Model evaluation (3-day prediction error)	0.294		2.032		0.214		3.146		0.427		0.396	
**3-day forecasting**													
	Cumulative difference	42		30		28		40		66		81	
	Accuracy (%)	98.7		85.1		93.3		69.0		76		72.2	
**7-day forecasting**													
	Cumulative difference	670		110		147		45		175		144	
	Accuracy (%)	90.9		76.7		85.2		85.9		68.3		76.8	
**14-day forecasting**													
	Cumulative difference	2858		272		541		217		452		329	
	Accuracy (%)	81.6		72.1		74.5		72.6		60.3		73.6	

### COVID-19 Daily New Cases Forecasting

Table 1 also presents the results of forecasting and prediction accuracy. Using the final models with the selected time windows, COVID-19 daily new cases were forecasted for the next 14 days at both the state and county level. At the state level, the 3-day cumulative difference and prediction accuracies were 42 and 98.7%, respectively. As compared to the 3-day prediction accuracy, the 7- and 14-day accuracies reduced to 90.9% and 81.6%, respectively. At the county level, among the top five counties, the 3-day prediction accuracy ranged from 69.0%-93.3%. The prediction accuracy deceased in Charleston, Greenville, and Spartanburg with increased time span. In contrast, the prediction accuracy in Horry and Richland increased in the 7-day prediction but decreased in the 14-day prediction. The 14-day prediction accuracies for Horry and Richland were closer to their values in the 3-day prediction. Table S2 in Multimedia Appendix 1 presents the predicted and observed cases of COVID-19 in the final models.

## Discussion

### Principal Findings

This study leveraged disease surveillance data and Twitter-based population mobility data to test the relationship between mobility and COVID-19 daily new cases and forecast transmission during the next 14 days at both the state and county level in South Carolina. Results revealed that population mobility was significantly and positively associated with new daily COVID-19 cases. Using the selected models to forecast COVID-19 transmission, we found that although the prediction accuracy at the state level and most of the selected counties decreased as the time span increased, the prediction accuracy remained acceptable. To the best of our knowledge, this is the first study that combined correlation analysis and forecasting together to investigate the impacts of population mobility on COVID-19 transmission at both the state and county level.

Population mobility could reflect the impacts of NPIs, reopening policies, and public holidays, and estimate social movement during the current COVID-19 pandemic. It is closely related to COVID-19 outbreaks, which is in accordance with the findings of prior research [9,11,17-19]. This study adds value to previous studies by examining the impacts of population mobility on COVID-19 incidence at both the state and county level in South Carolina. The results revealed a positive association of population mobility with daily new COVID-19 cases. However, it should be noted that the population mobility data used in our study only reflected the mobility of people who used Twitter, although such mobility data have been validated to be a good proxy of actual human movement during the pandemic [16,26,27]. Additionally, those Twitter users tended to be young, which might influence how much and what they tweet. The sociodemographic characteristics of Twitter users may be potential confounders, which were not controlled for in our study. Thus, caution is needed when interpreting our findings. Future studies are needed to consider and control for the sociodemographic characteristics of Twitter users.

Using Twitter-based mobility data to predict daily new COVID-19 cases could yield acceptable accuracy, which could also justify the prediction efficacy of this indicator. The high prediction accuracy at the state level was consistent with Wang’s finding in Arizona [19]. However, such a high prediction accuracy was not found at the county level. One possible explanation for this finding is that we did not capture or account for the influences of contextual factors (ie, population density) and the roles of mitigating factors (eg, wearing a face mask, practicing social distancing) [18,19,28]. Additionally, the Twitter-based mobility data did not differentiate between social movement at different locations, such as parks, workplaces, and retail locations, which have different impacts on COVID-19 incidence [9]. Finally, in this study, we only captured population mobility at the state and county level, while population mobility at the zip code level might provide a more accurate prediction. Nevertheless, the findings generated from our study confirmed the spatial-temporal relationship between Twitter-based mobility and COVID-19 outbreaks in South Carolina and the acceptable prediction efficacy of population mobility.

Our findings provide empirical evidence to support the application of Poisson count time series and time-varying population mobility data in improving the accuracy of COVID-19 forecasting. Compared with the existing literature, our models yielded acceptable prediction accuracy for two-week forecasting at both the state and county level. Time-varying population mobility could be incorporated into other forecasting models, such as classic time series methods and machine learning [22,24]. Since we are particularly interested in count data, we preferred the Poisson count time series model. When modelling rate, ARIMA and Holt-Winters are more appropriate than the Poisson count time series model. Regarding machine learning, most models are applied to the prediction of binary or categorical variables, and future studies are needed to apply them to predicting the count outcome with time-varying population mobility.

The use of population mobility data has potential implications for future research and practices to curb COVID-19 outbreaks. From a research perspective, research on mobility and COVID-19 could be studied at the state, county, and/or zip code level. In addition, mobility around different locations could provide detailed information regarding COVID-19 transmission, identify the most relevant mobility associated with daily new cases, and inform tailored interventions on social distancing by location to control disease outbreaks. Furthermore, the geospatial difference in the prediction accuracy of population mobility for daily new cases by county suggested that contextual factors—such as demographic characteristics and the implementation fidelity of NPIs at the county level—should be accounted for in future research. Finally, since the incubation and transmission of COVID-19 are closely associated with time-varying factors, such as temperature and weather, such impacts should be accounted for in forecasting studies [29]. Regarding the practice of disease control and prevention, leveraging social media platforms to monitor daily population mobility could improve predictions of further COVID-19 transmission, inform proactive NPIs, and guide the allocation of health care resources to reduce disease morbidity and mortality [30,31].

### Conclusions

Population mobility was positively associated with COVID-19 transmission at both the state and county level in South Carolina. Using Twitter-based mobility data could enable acceptable predictions of COVID-19 daily new cases. The use of social media data to monitor population mobility and predict COVID-19 spread could inform proactive measures to curb disease outbreaks and plan coordinated responses.

## Appendix

Multimedia Appendix 1 Supplementary data.

## Abbreviations

ARIMA: autoregressive integrated moving average
NPI: nonpharmaceutical intervention
