# Supplementary material for: Spatial-Temporal Relationship Between Population Mobility and COVID-19 Outbreaks in South Carolina: Time Series Forecasting Analysis
Source: J Med Internet Res. 2021 Apr 13;23(4):e27045. doi: 10.2196/27045 (PMC8045774; doi:10.2196/27045)
Supplement: Multimedia Appendix 1 [file jmir_v23i4e27045_app1.docx]

Table S1. Specification table

| **Subject** | **Infectious diseases** |
| --- | --- |
| Specific subject area | Count time series following generalized linear models applied to assess the prediction efficacy of Twitter-based population mobility on daily COVID-19 new cases at both state and county level in South Carolina |
| Type of data | R script file  CSV data file |
| How data were acquired | COVID-19 case data were acquired through “us-counties.csv” and “us-states.csv” obtained from The New York Times (https://github.com/nytimes/covid-19-data) between March 6 and November 11, 2020. This provides cumulative COVID-19 case data up to November 11, 2020.  Twitter-based population mobility was obtained through Origin-Destination-Time Flow Explorer (http://gis.cas.sc.edu/GeoAnalytics/od.html) from March 6 to October 31, 2020. This dataset provides numbers of Twitter users who posted at least twice a day or posted tweets on at least two consecutive days.  Forecasting was conducted using Poisson count time series model.  Rstudio version 1.1.456 and R version 3.6.3 were employed to generate the code and perform data analysis. The R-packages “tscount”, “ggplot2”, “forecast”, “cowplot”, “gtable”, “grid”, “gridExtra”, “anytime”, “dplyr”, and “lubridate” are required to run the codes and generate the figures. |
| Data format | Data were raw, filtered, and analyzed.  R script file (code) and data were executed in RStudio. |
| Description of data collection | Using the cumulative COVID-19 case count data at both state and county level from The New York Times, we filtered for “South Carolina” and obtained all the cases from March 6 to November 11, 2020. Daily incidence was calculated by subtracting the cumulative confirmed cases of previous day from the total cases for the entire state and its five counties with largest numbers of cumulative confirmed cases (i.e., Charleston, Greenville, Horry, Spartanburg, and Richland).  Population mobility was extracted from the relevant platform. The mobility at state and county level in South Carolina was used for data analysis. |
| Parameters | State and county level forecasting are performed using daily COVID-19 new cases and population mobility. Count time series model with Poisson distribution was used for analysis. Log link function was specified in the time series model. Forecast lengths include three, seven, and fourteen days forward. Lookback lengths included one, seven, fourteen and all prior days.  Prediction error, cumulative difference, and mean absolute percentage accuracy were calculated to evaluate the model performance. |
| Data source location | University of South Carolina – Geoinformation and Big Data Research Laboratory, Columbia, SC, USA |
| Data accessibility | The data is hosted in a public repository and entitled: “Twitter-based population mobility and COVID-19 forecasting in South Carolina R-script and data” and made available through the platform.  Repository name: Mendeley Data  Direct URL to data and script: doi:10.17632/68chgz6chh.1  Instruction for accessing these data and script:  Access, download, and extract the data resource and script from the URL. Using RStudio to open the R script "Mobility and COVID-19 in SC". Change the path manually and import the data resources. This script included the data management and analysis. No additional changes are needed. Run the script based on the instruction. In terms of the forecasting for specific county, users need to change the name of county manually. |

Table S2. Descriptive statistics of population mobility and COVID-19 new cases at both state and county level

|  | **Minimum** | **25^th^ percentile** | **50^th^ percentile** | **75^th^ percentile** | **Maximum** |
| --- | --- | --- | --- | --- | --- |
| **Population mobility** |  |  |  |  |  |
| **State level** |  |  |  |  |  |
| March | 658 | 809 | 1,010 | 1,109 | 1,438 |
| April | 554 | 617 | 670 | 697 | 786 |
| May | 630 | 754 | 812 | 848 | 940 |
| June | 756 | 848 | 871 | 910 | 993 |
| July | 818 | 870 | 896 | 937 | 1,039 |
| August  September  October | 767  784  789 | 828  831  843 | 863  875  898 | 884  907  965 | 1,035  1,021  1,085 |
| **County level** |  |  |  |  |  |
| **Charleston** |  |  |  |  |  |
| March | 81 | 104 | 126 | 142 | 195 |
| April | 62 | 75 | 83 | 92 | 98 |
| May | 73 | 93 | 104 | 121 | 140 |
| June | 96 | 109 | 116 | 126 | 154 |
| July | 95 | 109 | 117 | 121 | 133 |
| August  September  October | 88  94  95 | 99  110  113 | 109  116  122 | 120  126  132 | 134  150  142 |
| **Greenville** |  |  |  |  |  |
| March | 103 | 115 | 139 | 156 | 177 |
| April | 82 | 93 | 106 | 114 | 134 |
| May | 100 | 113 | 119 | 127 | 132 |
| June | 104 | 117 | 124 | 133 | 162 |
| July | 107 | 124 | 135 | 140 | 153 |
| August  September  October | 111  114  104 | 129  128  133 | 140  140  138 | 146  144  149 | 168  158  169 |
| **Horry** |  |  |  |  |  |
| March | 77 | 84 | 87 | 116 | 158 |
| April | 53 | 64 | 71 | 80 | 97 |
| May | 76 | 87 | 100 | 128 | 151 |
| June | 103 | 113 | 125 | 133 | 162 |
| July | 100 | 116 | 123 | 140 | 171 |
| August  September  October | 89  79  71 | 112  96  99 | 118  107  105 | 137  117  116 | 160  143  151 |
| **Spartanburg** |  |  |  |  |  |
| March | 40 | 67 | 82 | 89 | 106 |
| April | 34 | 43 | 47 | 50 | 61 |
| May | 47 | 51 | 56 | 62 | 72 |
| June | 50 | 62 | 65 | 72 | 78 |
| July | 51 | 67 | 76 | 85 | 101 |
| August  September  October | 50  55  52 | 65  62  59 | 70  65  67 | 77  70  79 | 94  74  92 |
| **Richland** |  |  |  |  |  |
| March | 58 | 76 | 82 | 93 | 120 |
| April | 53 | 68 | 73 | 78 | 84 |
| May | 61 | 69 | 77 | 84 | 115 |
| June | 65 | 77 | 86 | 93 | 105 |
| July | 59 | 76 | 82 | 95 | 105 |
| August  September  October | 72  72  72 | 79  82  84 | 89  89  92 | 95  97  100 | 109  125  119 |
| **COVID-19 new cases** |  |  |  |  |  |
| **State level** |  |  |  |  |  |
| March | 0 | 3 | 18 | 74 | 158 |
| April | 62 | 131 | 154 | 204 | 275 |
| May | 82 | 129 | 164 | 228 | 467 |
| June | 236 | 476 | 757 | 1,115 | 1,755 |
| July | 972 | 1,520 | 1,726 | 1,855 | 2,374 |
| August  September  October | 456  301  381 | 722  624  789 | 937  863  912 | 1,214  1,190  1,057 | 1,583  2,665  1,706 |
| **County level** |  |  |  |  |  |
| **Charleston** |  |  |  |  |  |
| March | 0 | 0 | 1 | 8 | 32 |
| April | 0 | 3 | 5 | 12 | 48 |
| May | 0 | 1 | 6 | 8 | 23 |
| June | 11 | 34 | 69 | 200 | 373 |
| July | 85 | 164 | 221 | 303 | 418 |
| August  September  October | 25  0  13 | 53  35  34 | 95  46  50 | 105  65  61 | 218  425  89 |
| **Greenville** |  |  |  |  |  |
| March | 0 | 1 | 5 | 11 | 18 |
| April | 0 | 9 | 19 | 28 | 54 |
| May | 7 | 14 | 21 | 33 | 150 |
| June | 47 | 71 | 115 | 147 | 245 |
| July | 49 | 129 | 167 | 196 | 276 |
| August  September  October | 14  6  27 | 40  41  87 | 53  75  107 | 95  113  140 | 184  289  197 |
| **Horry** |  |  |  |  |  |
| March | 0 | 1 | 2 | 3 | 5 |
| April | 0 | 2 | 5 | 9 | 18 |
| May | 0 | 4 | 5 | 10 | 26 |
| June | 17 | 47 | 99 | 133 | 221 |
| July | 63 | 103 | 145 | 189 | 358 |
| August  September  October | 16  4  15 | 30  20  48 | 41  30  73 | 56  46  90 | 115  70  139 |
| **Spartanburg** |  |  |  |  |  |
| March | 0 | 0 | 0 | 2 | 7 |
| April | 1 | 4 | 6 | 11 | 32 |
| May | 1 | 4 | 7 | 14 | 61 |
| June | 5 | 18 | 34 | 44 | 72 |
| July | 18 | 48 | 63 | 84 | 125 |
| August  September  October | 11  2  0 | 25  18  46 | 44  50  78 | 62  99  96 | 92  215  147 |
| **Richland** |  |  |  |  |  |
| March | 1 | 3 | 6 | 14 | 37 |
| April | 3 | 15 | 25 | 32 | 56 |
| May | 5 | 15 | 19 | 26 | 33 |
| June | 12 | 44 | 67 | 81 | 155 |
| July | 57 | 108 | 138 | 165 | 234 |
| August  September  October | 39  34  24 | 79  77  51 | 93  96  67 | 124  142  78 | 408  766  130 |

Table S3. The predicted and observed cases of COVID-19 in the final models

|  | **State level** |  | **County level** | | | | |
| --- | --- | --- | --- | --- | --- | --- | --- |
|  |  | **Charleston** | | **Greenville** | **Horry** | **Spartanburg** | **Richland** |
| **Forecasting** |  |  | |  |  |  |  |
| Prediction |  |  | |  |  |  |  |
| 238^th^ day | 1,097 | 64 | | 128 | 71 | 85 | 62 |
| 239^th^ day | 1,031 | 68 | | 135 | 43 | 106 | 76 |
| 240^th^ day | 1,029 | 69 | | 130 | 53 | 59 | 72 |
| 241^st^ day | 1,091 | 67 | | 142 | 51 | 78 | 72 |
| 242^nd^ day | 1,034 | 74 | | 160 | 34 | 83 | 66 |
| 243^rd^ day | 1,073 | 73 | | 130 | 67 | 64 | 75 |
| 244^th^ day | 1,049 | 79 | | 149 | 41 | 69 | 70 |
| 245^th^ day | 1,096 | 82 | | 138 | 44 | 100 | 74 |
| 246^th^ day | 1,085 | 85 | | 149 | 48 | 88 | 80 |
| 247^th^ day | 1,096 | 88 | | 140 | 39 | 81 | 78 |
| 248^th^ day | 1,105 | 91 | | 146 | 47 | 78 | 78 |
| 249^th^ day | 1,104 | 94 | | 150 | 38 | 71 | 78 |
| 250^th^ day | 1,113 | 98 | | 149 | 35 | 64 | 81 |
| 251^st^ day | 1,114 | 101 | | 147 | 49 | 80 | 80 |
| Observation |  |  | |  |  |  |  |
| 238^th^ day | 1,100 | 78 | | 133 | 47 | 89 | 92 |
| 239^th^ day | 1,003 | 54 | | 155 | 42 | 86 | 122 |
| 240^th^ day | 1,018 | 71 | | 127 | 39 | 101 | 76 |
| 241^st^ day | 1,411 | 96 | | 186 | 49 | 124 | 123 |
| 242^nd^ day | 894 | 49 | | 138 | 36 | 48 | 62 |
| 243^rd^ day | 1,035 | 67 | | 92 | 67 | 61 | 72 |
| 244^th^ day | 918 | 59 | | 164 | 43 | 45 | 76 |
| 245^th^ day | 769 | 57 | | 63 | 51 | 22 | 87 |
| 246^th^ day | 1,233 | 63 | | 159 | 54 | 152 | 73 |
| 247^th^ day | 1,870 | 101 | | 299 | 94 | 165 | 124 |
| 248^th^ day | 946 | 77 | | 121 | 47 | 69 | 65 |
| 249^th^ day | 703 | 49 | | 107 | 36 | 55 | 43 |
| 250^th^ day | 1,347 | 63 | | 200 | 101 | 59 | 83 |
| 251^st^ day | 1,257 | 93 | | 177 | 86 | 60 | 148 |
